# New Horizons for Hydroxyapatite Supported by DXA Assessment—A Preliminary Study

**DOI:** 10.3390/ma15030942

**Published:** 2022-01-26

**Authors:** Jakub Litak, Cezary Grochowski, Andrzej Rysak, Marek Mazurek, Tomasz Blicharski, Piotr Kamieniak, Piotr Wolszczak, Mansur Rahnama-Hezavah, Grzegorz Litak

**Affiliations:** 1 Department of Neurosurgery and Pediatric Neurosurgery, Medical University of Lublin, Jaczewskiego 8, 20-954 Lublin, Poland; cezary.grochowski@o2.pl (C.G.); marekmazurek@hotmail.com (M.M.); pkamieniak@poczta.onet.pl (P.K.); 2 Department of Automation, Faculty of Mechanical Engineering, Lublin University of Technology, Nadbystrzycka 36, 20-618 Lublin, Poland; rysak.andrzej@gmail.com (A.R.); p.wolszczak@pollub.pl (P.W.); g.litak@pollub.pl (G.L.); 3 Department of Rehabilitation and Orthopedics, Medical University of Lublin, ul. Jaczewskiego 8, 20-090 Lublin, Poland; blicharski@vp.pl; 4 Chair and Department of Oral Surgery, Medical University of Lublin, 20-090 Lublin, Poland; mansur.rahnama@umlub.pl

**Keywords:** DXA, density, hydroxyapatite

## Abstract

Dual Energy X-ray Absorptiometry (DXA) is a tool that allows the assessment of bone density. It was first presented by Cameron and Sorenson in 1963 and was approved by the Food and Drug Administration. Misplacing the femoral neck box, placing a trochanteric line below the midland and improper placement of boundary lines are the most common errors made during a DXA diagnostic test made by auto analysis. Hydroxyapatite is the most important inorganic component of teeth and bone tissue. It is estimated to constitute up to 70% of human bone weight and up to 50% of its volume. Calcium phosphate comes in many forms; however, studies have shown that only tricalcium phosphate and hydroxyapatite have the characteristics that allow their use as bone-substituted materials. The purpose of this study is aimed at analyzing the results of hip densitometry and hydorxyapatite distribution in order to better assess the structure and mineral density of the femoral neck. However, a detailed analysis of the individual density curves shows some qualitative differences that may be important in assessing bone strength in the area under study. To draw more specific conclusions on the therapy applied for individual patients, we need to determine the correct orientation of the bone from the resulting density and document the trends in the density distribution change. The average results presented with the DXA method are insufficient.

## 1. Introduction

Dual energy X-ray absorptiometry (DXA) is a tool that allows the assessment of bone density. It was first presented by Cameron and Sorenson in 1963 and was approved by the Food and Drug Administration in the US in 1988 [1,2]. This device, if widely used in clinical practice, allows the estimation of changes in bone density. It is the gold standard for measuring bone mineral density, and its results are also used in the calculation of fracture risk [3]. The beam with the two energy peaks is created in order to separate the bone from the soft tissue and spans a wide range of photon energies. It is essential that the beam must be produced in only two energy peaks. The diagnostic test measuring bone density in the proximal femur lasts only 1–2 min and allows the evaluation not only of the bone density changes during a disease process but also after pharmacologic intervention for the treatment of osteoporosis. Measurements should consider only spine or proximal femur tests, and, according to several studies, for instance Watts et al. [4,5], other skeletal sites by any different techniques are not recommended. In order to achieve worthy results and the proper functioning of the device, a daily calibration scan with a phantom is needed. This allows the correct results of software algorithms as well as a precise evaluation of shift or drift in bone mineral density (BMD) values [6,7] The other important factor ensuring the quality of DXA interpretation is the use of a representative clinical population and a precision study by technologists following certain guidelines in order to assess how much of a change is real [8,9].

Misplacing the femoral neck box, placing a trochanteric line below the midland and improper placement of boundary lines are the most common errors made during a DXA diagnostic test made by auto analysis [10]. It is essential to maintain the femoral neck parallel to the table and have the smallest cross-section of the femoral neck presented to the incident X-rays. To obtain this, the hip should be internally rotated, and the shaft of the femur should be positioned straight. Rotation of the hip should be avoided to maintain accuracy and precision. Rosenthall et al., in their study, revealed that transition of the hip from internal rotation to neutral rotation was linked to an increased BMD in the femoral neck [11].

Studies on the density of bone material can provide the knowledge necessary for the development of many fields of medicine. One of these is reconstructive tissue engineering, which is playing an increasingly important role in modern orthopedics and surgery. The use of artificial bone material is necessary in many conditions in which natural tissue must be removed as a result of infection, cancer or traumatic changes. In these cases, transplant appears to be the best solution, but this therapeutic option is not always available [12]. An alternative can be the use of synthetic grafts, designed to best reflect the characteristics of natural human tissue [13,14]. The materials used for this purpose must have a number of properties, such as: appropriate mechanical parameters, porosity of structure, neutrality for the body, the ability to create an appropriate environment and promote the regeneration of body tissues, as well as the non-toxicity and biocompatibility of degradation products [15]. Currently, many centers around the world are working intensively to develop the best solution for artificial bone material transplantation [13]. One of the materials with potential use in this role seems to be hydroxyapatite (HAp). It is a mineral with the formula Ca10(PO4)6(OH)2, being one of the forms of calcium phosphate (CaP) [16,17,18].

Unfortunately, none of the forms of CaP is ideal for medical applications, and its use has a number of restrictions [19,20]. For this reason, much attention is paid to modifying its structure to improve its biochemical parameters. Hydroxyapatite alone, despite the fact that it has unique features supporting the healing process, cannot be commonly used in the production of implants and cement alone due to its high fragility and low flexibility. Natural bone resembles a composite material made of HAp and collagen fibers [21]. Many centers try to modify the structure of hydroxyapatite with various polymers to better map the characteristics of natural tissue [22,23,24,25,26].

Hydroxyapatite is the most important inorganic component of teeth and bone tissue [17,27]. It is estimated to constitute up to 70% of human bone weight and up to 50% of its volume [22,23,28]. Calcium phosphate comes in many forms; however, studies have shown that only tricalcium phosphate and hydroxyapatite have the characteristics that allow them to be used as bone-substituted materials [12,13,22,29,30]. They are characterized by biomimetic morphologies and osteoconductive properties. In vivo studies have shown that, surrounded by an implanted synthetic graft composed of 70% hydroxyapatite and 30% calcium beta-phosphate, there are no differences in the distribution of osteoblasts and osteoclasts compared to natural bone tissue [31]. Additionally, in the work of De Godoy et al. [32], the high efficiency of biomaterial-based integration of hydroxyapatite particles into bone has been demonstrated, resulting in a low incidence of postoperative complications. Moreover, the presence of hydroxyapatite supports the natural processes of bone formation and tissue vascularization [33,34]. This is caused by the fact that it is a suitable scaffold for the formation of a fibrin network and that it has a high cell conductivity [35,36]. Interestingly, these properties do not only refer to the local supply of hydroxyapatite. Oral administration has also shown a positive effect on the healing and repair of damaged bone as well as the prevention of osteoporosis [37]. These properties allow the use of HAp among others, specifically as a drug-delivery system for bone therapy, with spinal fusion, bone augmentations, bone fillers after tumor surgery, maxillofacial reconstruction or artificial bone grafts [17,24,38,39,40,41,42].

The purpose of our study is aimed at analyzing the results of hip densitometry and hydorxyapatite distribution in order to better assess the structure and mineral density of the femoral neck. It is based on the numerical analysis of digital images obtained using a factory-produced densitometer. In the proposed approach, the information contained in the rentgenogram pixel values, distributed in the rectangular area crossing the femoral neck, is transformed into a mathematical curve showing the approximate distribution of matter density in this area. In the field of densitometry, we will focus on a simple task, which is to compare the results obtained numerically for a group of several patients. The preliminary results obtained are promising and allow the supposition that the development of the proposed method can provide an effective tool for the numerical assessment of matter density within the area of the femoral neck.

Our study also presents literature review systematizing knowledge about the use of hydroxyapatite in modern therapy.

## 2. Materials and Methods

The analyzed densitometric results were randomly selected from a group of numerous standard tests. For simplicity, the individual results have been named according to the successive letters of the alphabet, from A to H (eight studies). All studies were carried out under standard and repeatable conditions. The patients signed formal consents for participation in the study, applied with the standard procedure. The apparatus (GE Healthcare; The Prodigy^®^ Advance) used had the necessary approvals and was regularly calibrated in accordance with the manufacturer’s instructions. Processed images are used for diagnostic purposes. Based on them, we want to propose a numerical approach that can bring additional information to the analysis of the result. Its special feature is the presentation of density changes along a rectangular area that crosses the femoral neck. Of course, the analysis of raw data obtained from densitometric measurement can bring refinement of results, but their qualitative changes are expected to be very similar. All patients received written and verbal information regarding the study design and procedures. Signed informed consent was obtained from all participating patients. The study protocol and informed consent forms were approved by the Ethics Committee of the Medical University of Lublin in Poland in accordance with binding legislation.

### Numerical Procedure

**Figure 1:**
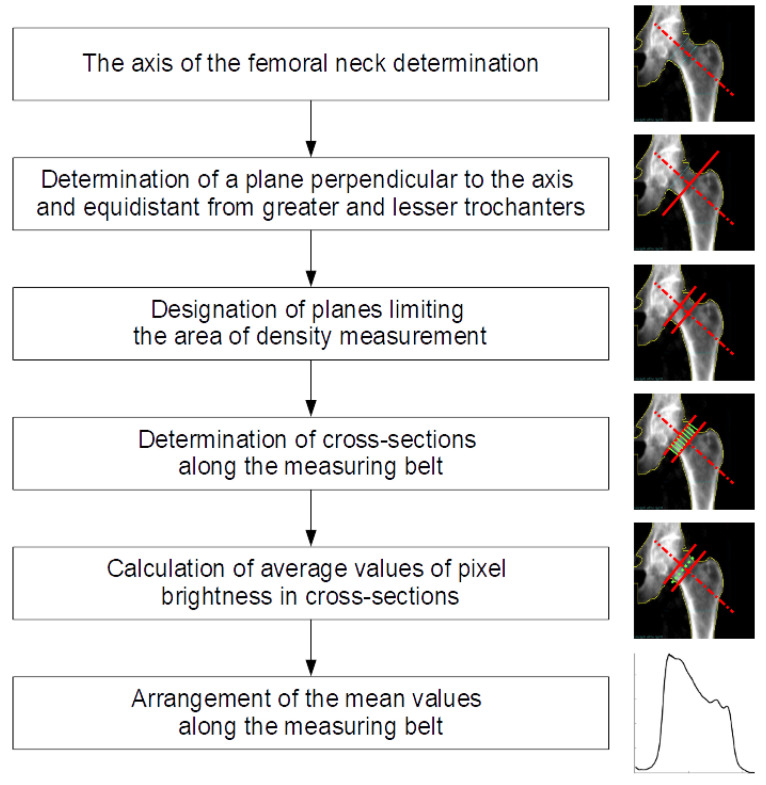
Schema of image processing and bone density measurements.

In the analyzed black and white images, areas with higher X-ray absorption are marked with lighter pixels. We assume that the brightness of a given area is proportional to the density of the material in the volume defined by the projection of the given surface in the area between the source and the radiation detector. As a result of a number of numerical operations, from a single study we obtain a curve that approximately represents changes in bone mineral density determined along the “cross-sectional belt’’—a rightly defined rectangular area crossing the femoral neck. We call this curve the femoral neck density curve. The position of the cross-sectional belt on the surface of the densitometric image is determined using technical reference lines automatically added to the test result by the diagnostic device. Ultimately, we intend to develop our own positioning of the cross-sectional area in the femoral neck, but at this point we want to be able to compare new results with the results obtained from the measurement system. In this paper, we briefly describe the method of deriving density curves and show the preliminary results of comparing the density curves calculated for selected densitometric tests. In fact, the pixel pattern of the image roughly corresponds to the bone mineral density. The distribution and brightness (values) of pixels depend on the technical implementation of the measurement and the interpretation methods implemented by the device and software manufacturer. Numerical analysis begins with the conversion of the graphic measurement result into a numerical matrix with natural values and dimensions determined by the pixel resolution of the image being examined. Figure 1 shows schema of image processing and bone density measurements.

**Figure 2:**
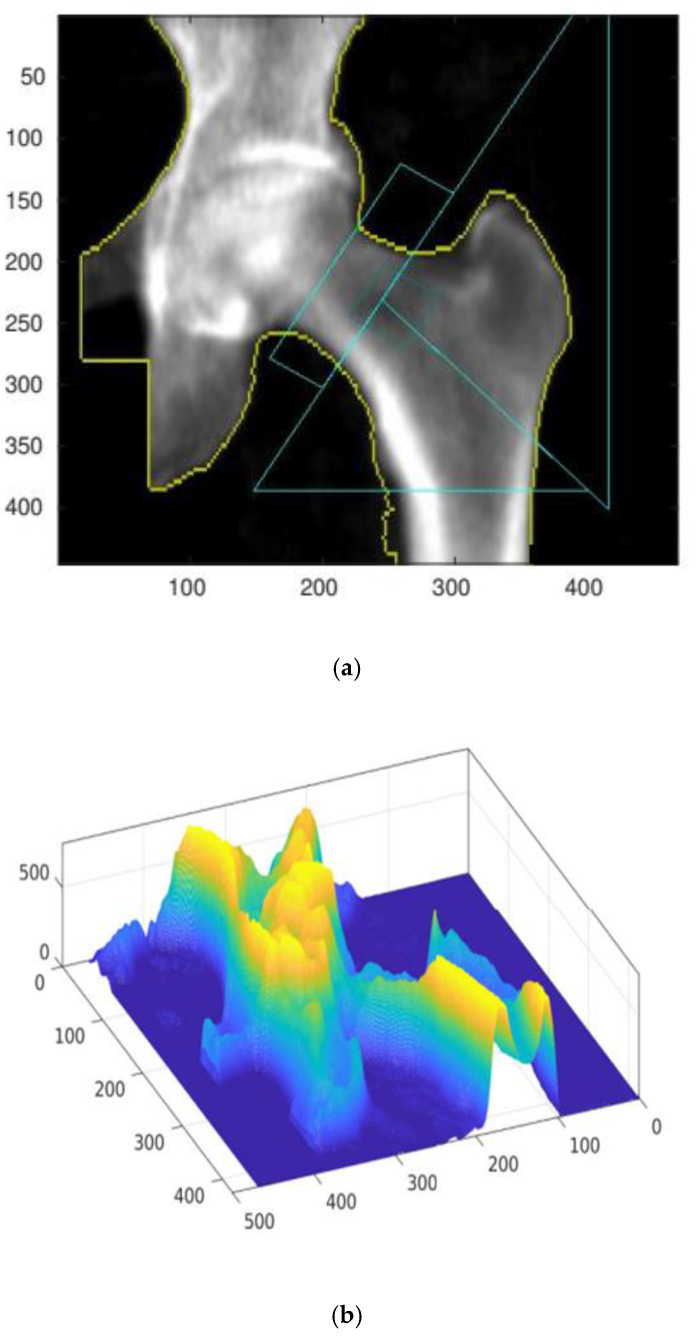

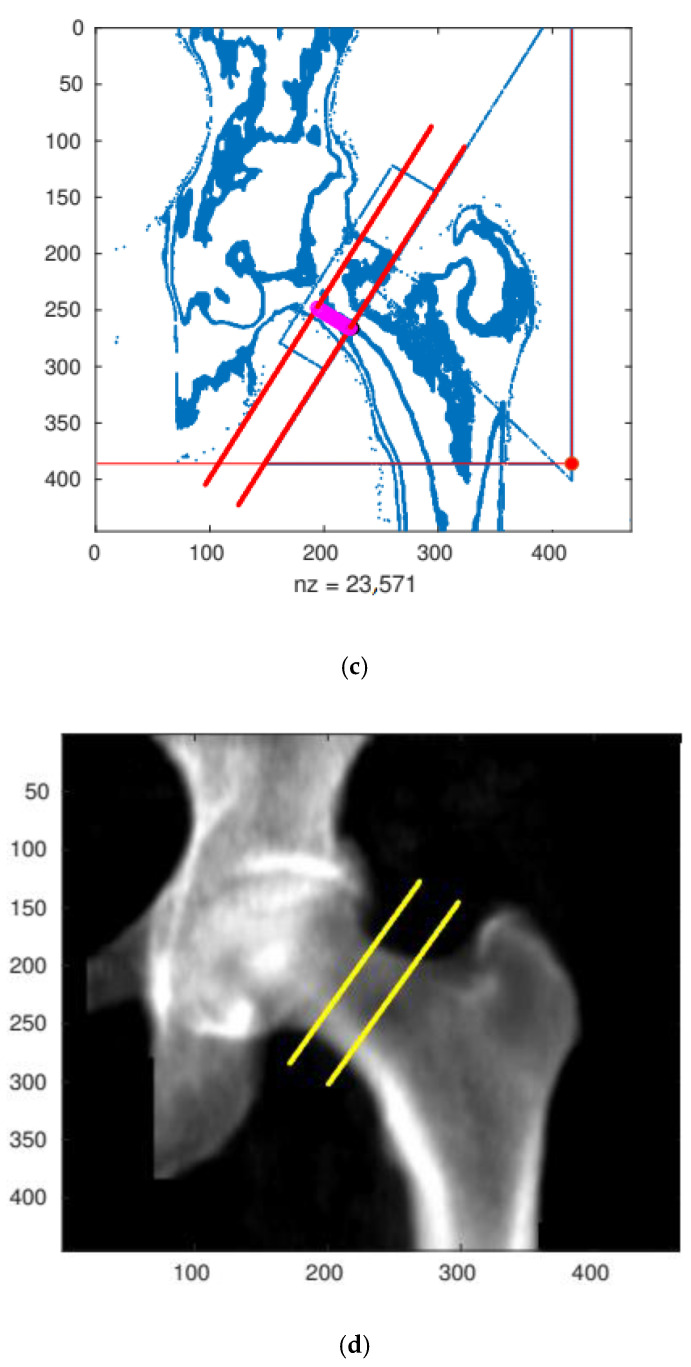
The result of the densitometry test with automatically added reference lines (**a**) and the corresponding numerical matrix defining density distribution depending on the projection plane position (**b**). Identification of the focusing cross-sectional belt zone (**c**,**d**) for numerical evaluation. All presented results were measured and calculated for the A result.

The numeric matrix can be presented on a 3D graph, assuming that the variables *x*, *y* correspond to the coordinates of a given pixel, and assigning the variable *z* a value corresponding to its brightness. Figure 2 presents a comparison of the starting densitometric image (a) with the corresponding numerical matrix (b).

The numerical procedure first determines the positions and slopes of individual reference lines, drawn automatically by the densitometer measuring system (GE Lunar Prodigy). The correct operation of the algorithm is verified by displaying control plots. An exemplary control plot is shown in Figure 2c.

After identifying the reference lines, the area of the cross-sectional belt is determined, which in Figure 2d is marked with two yellow boundary lines. The direction and position of the right-bottom yellow boundary line defines the main analysis line (MAL) along which the approximate bone density value is calculated.

## 3. Results

The density curve is created by moving the detection line segment along the cross-sectional belt, the former of which is perpendicular to both boundary lines, while its length is equal to the distance between them.

Estimation of bone density along the section belt is calculated by averaging the values of all pixels located on the sliding detection line segment.

**Figure 3:**
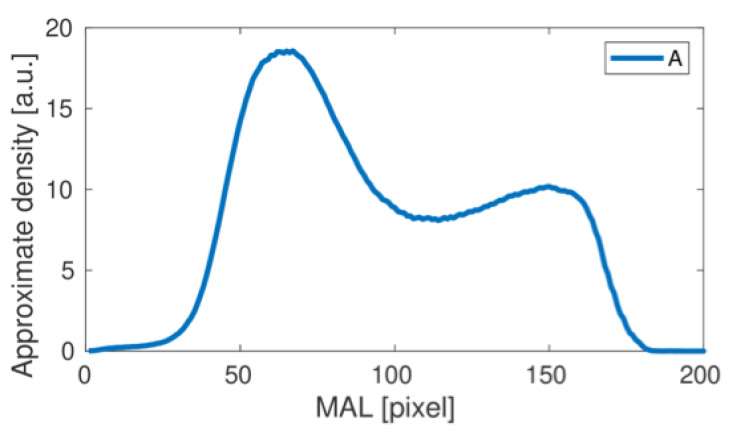
The femoral neck density curve obtained for the A study. Pixels correspond to length units.

The femoral neck density curve determined for the results presented in Figure 2 (A test) is shown in Figure 3.

This is a typical result obtained using the proposed numerical analysis. The first wide bone density peak corresponds to the high density area at the bottom of the cross-sectional band on the inferior part (inferior cortical bone) of the femoral neck in the images presented above.

**Figure 4:**
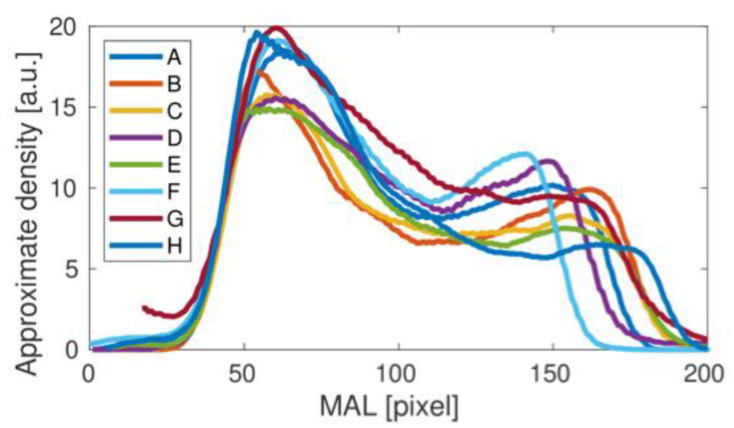
Femoral neck density curves obtained for all of the analyzed studies (A–H).

A second, much weaker density peak corresponds to a slight increase in bone density on the superior part of the femoral neck-superior cortical bone. By repeating the numerical procedure for subsequent studies, we obtain the complete set of density curves shown in Figure 4.

**Figure 5:**
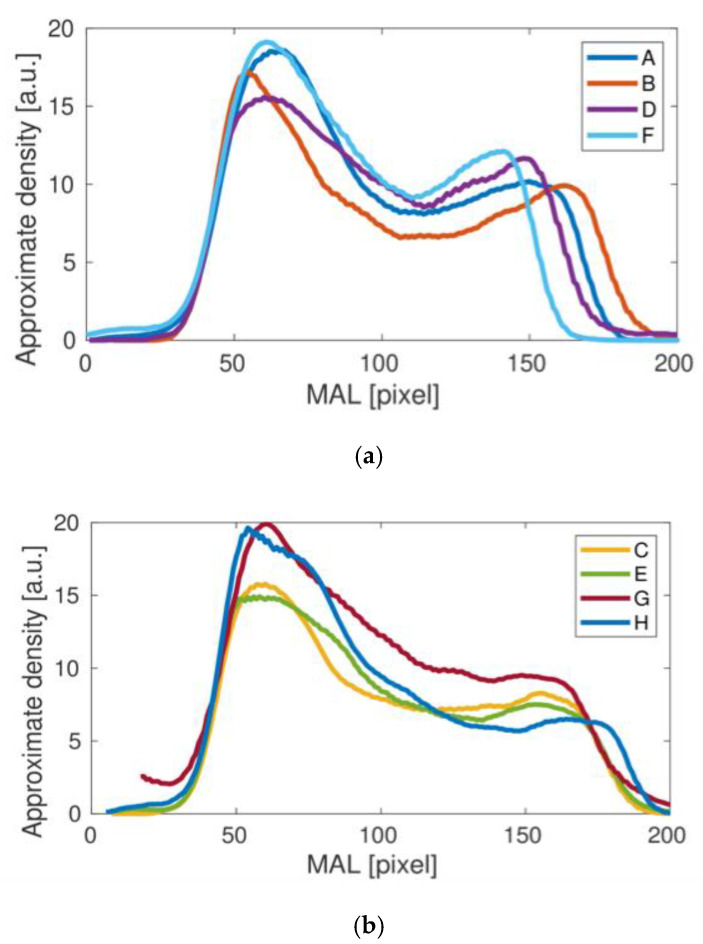
Femoral neck density curves obtained for the studies A, B, D, F (**a**) and C, E, G, H (**b**) clearly differ qualitatively.

Comparing the results of the density curves obtained for all tests, it can be seen that their qualitative variability is similar to that shown in Figure 4 for the result A. The differences in the height and width of the density curves result directly from the different size of bones measured in individual studies. However, a detailed analysis of the individual density curves shows some qualitative differences that may be important in assessing bone strength in the area under study. To better illustrate the differences observed, the results obtained were divided into two groups, presented in Figure 5. For the upper panel, the middle dip in bone density is clear. In the lower panel this dip does not appear. The main cause could be related to the fairly small height of the second density peak. Interestingly, the length of the density distribution varies in the upper panel of Figure 5, but it stabilizes in the lower panel as the longest. It could be related to the special orientation of the bone during the medical treatment. Besides, one can also notice a number of individual differences between the studied cases C, E, G and H, including heights.

Table 1 shows the values of the local extremes of exemplary brightness curves corresponding to bone density. The “maximum 1” value corresponds adequately to the density in area of the greater trochanter and “maximum 2” adequately to the lesser trochanter. The “Minimum 1” value is the central local minimum of the curve corresponding to the area between the trochanters.

The table contains a relative decrease value, calculated as the difference between the brightness of the minimum 1 and the maximum 2 in relation to this value expressed as a percentage. The difference between the group means of 14.5% and 2.2% with low values of standard deviation confirms the observations presented above.

## 4. Discussion

### 4.1. Hydroxyapatite Matter

In recent years, many researchers have also focused on developing new materials based on nanoparticle technology. Until now, they have been used in, among other things, gene-delivery systems and drug production, as well biosensors, catalysts and optical devices [43,44]. Currently, many centers are working on the creation of hydroxyapatite nanoparticles (HApN) and their use in biomedical applications [13,38,39,45]. The technology of materials based on nanostructures is based on the concentration of many particles on the surface of a material [21]. This form of distribution allows you to bypass the limitations of conventional methods and gives new properties compared to the standard HAp. The main advantage of hydroxyapatite nanoparticles is their larger surface area [46]. It is estimated that the transition from micrometric size particles to nano scale allows an increase in the contact surface of about 30–50% [47]. This feature has a positive effect directly on the biocompatibility of material. This is confirmed by both tests performed on bacterial cultures, as well as tests carried out in vivo [48,49]. Additionally, in observations carried out in vivo by Brum et al., it was shown that the synthetic composite material consisting mainly of hydroxyapatite nanoparticles (78.76%) is characterized by close to zero cytotoxicity. Moreover, the authors also noted that the biggest advantage of the grafts created from HApN is the impact on the natural bone metabolism. At the insertion site, greater bone formation and new blood vessel formation have been demonstrated [47]. Earlier, the absorption of nanohydroxyapatite particles by osteoblasts has been repeatedly observed [50]. The unique regenerative properties of bone tissue under the influence of HApN have also been emphasized by Rossi et al. [51]. Similar observations have also been made by other authors investigating the effect of using bone cements based on hydroxyapatite nanoparticles in an animal model [52,53,54,55]. Sahana et al. showed that the intravenous supply of hydroxyapatite nanoparticles also has a positive effect on bone economy and the mechanical properties of bones [56].

Another advantage of hydroxyapatite-based materials is their ability to combine with other components. This allows the better reproduction of the characteristics of real human bone, which is also a composite material made of hydroxyapatite and collagen fibers [12,21,57]. In studies conducted by Zhang et al., the therapeutic effect of implanting bone implants in an animal model was checked. Researchers compared the results of treatment of pure chitosan grafts with composite grafts containing chitosan and nanohydroxyapatite. The results showed that in the case of a composite implant, both the bone regeneration period as well as the effectiveness of the implemented treatment was greater than in the second of the studied groups. This shows the undoubted effectiveness of this type of material in the regeneration of lost bone [58].

Modification of the materials based on nanotechnology in order to better carry out the functions of bone cement can also be achieved by other methods. One of them is the substitution of part of the atoms in the structure of the nanocrystals. Noor et al. conducted research on the Indonesian population checking the effect of the difference in atomic distribution in hydroxyapatite crystals on the density and porosity of bone material. They showed that the prevalence of some elements has a positive effect on the physicochemical properties of bones while in others this effect is negative [59]. In subsequent works, the influence of substitution of some atoms in the structure of hydroxyapatite nanocrystals on these parameters was examined. Landi et al. showed better properties of bone material while enriching the crystal structure with an additional amount of magnesium. This has also been confirmed in animal tests. Similar observations were made in the case of, among others, zinc, fluoride, silicon, carbonate, chloride, strontium and iron [60,61,62,63,64,65,66]. The proper composition of bone material is especially important for people suffering from osteoporosis. As mentioned earlier, this is characterized by a decrease in bone density and strength as a result of the chronic aggravation of bone architecture associated with a disproportion of osteoblast and osteoclast activities [67,68,69,70,71]. With an aging population, it is a more serious challenge to modern medicine [72,73,74]. It has been shown that in the bone tissue of people suffering from osteoporosis, there is a disturbance in the concentration of some minerals, which negatively affects the appropriate proportions of bone formation and bone resorption. It has been shown, inter alia, to reduce the titter of elements such as Sr, Fe, Zn, Mg, Mn, Ag, Pd, Na, K, P, Ca, Cr, Se and Cu [59]. In addition, osteoporotic bone also differs in the size and density of its bone crystal distribution compared to normal tissue [75]. The appropriate substitution of atoms in hydroxyapatite nanoparticles can positively influence the physicochemical parameters of bones, preventing pathological fractures. The beneficial effects of such a use of HAp have been demonstrated in, among others, studies on the prevention of postmenopausal bone loss, carried out by Castelo-Branco et al. [76]. They compared the effect of administering ossein hydroxyapatite with the administration of calcium carbonate. Long-term results have shown that administration of calcium carbonate is associated with a significant decrease in bone mass, whereas, in the case of ossein hydroxyapatite, this process did not occur, and bone mass was close to baseline [77]. Another study analyzing bone formation markers also showed a positive effect of HAp administration on bone turnover in women over 65 with densitometric osteoporosis of the lumbar spine or femoral neck [78].

Modifying the structure of crystals is also important for biological properties. Crystal phase, morphology and shape, as well as surface characteristics and particle size have been shown to condition cellular response [79,80]. This is due to the mechanism of the adsorption of nanoparticles by cells. This process occurs primarily as a result of endocytosis. In observations carried out by Russmueller et al., the presence of hydroxyapatite nanoparticles in the phagosomes of the analyzed osteoclasts was proved. Moreover, the authors suggested that the effect of this was the precipitation of calcium phosphate as part of the physiological control of cellular transport of calcium ions [81]. In other studies, Yin et al. showed that smaller particle size has a positive effect on the ease of absorption of hydroxyapatite nanoparticles by cells. Interestingly, the shape of the molecules also influenced this process. Rod-like particles were predisposed compared to those of a spherical shape [82]. The characterization of nanoparticle surfaces is also of great importance for the biological activity of cells. In his work, Liao et al. showed that the presence of surface defects can induce protein adsorption disorders by conditioning a weaker cellular response [83]. Some authors also emphasize the role of porosity and surface roughness in influencing the degree of cell proliferation [84,85]. The first of these features plays a key role in the conduction and bone remodeling process [34]. Bone cell transport through the pores of artificial bone material under the influence of metalloproteinases and inflammatory mechanisms contributes to the synthesis of new particles of natural hydroxyapatite, leading to bone regeneration [86]. Porosity also affects the physicochemical properties of the synthetic material itself, such as greater wettability [87]. Another parameter describing the surface condition of nanoparticles is their electric charge. Observations carried out in recent years indicate that the negative values of its charge cause greater adhesion of nanoparticles to bone cells, leading to their better absorption and more intense biological activity [88,89,90]. In the case of hydroxyapatite nanoparticles, the negative charge probably results from the presence of hydroxyl groups and phosphates in the HAp structure. This feature is also important due to HApN antiaggregation tendencies and in vitro particle stability [91,92,93].

The ability of hydroxyapatite nanoparticles to reach bone cells can be used not only to promote their metabolism. The human bone is a material relatively isolated from the rest of the body when it comes to drug distribution. The use of hydroxyapatite as a carrier particle could allow more effective treatment of many inflammatory diseases as well as bone and bone marrow cancers [91]. An example of the use of this feature is osteoimplants containing antibiotics [94,95]. Imaging diagnostics is another area in which this property could be used. Adding materials that facilitate imaging to the hydroxyapatite particles could allow for more accurate analysis of imaging diagnostics [91].

However, such good biodistribution of hydroxyapatite nanoparticles can also cause negative effects on the body, associated with the accumulation of material outside bone tissue. The abnormal distribution of Hap has previously been observed in Hap pathologies such as calcific tendinitis [96]. Studies conducted in an animal model showed a relatively large uptake of hydroxyapatite nanoparticles by the lungs. In other observations, increased aggregation in the spleen and liver was also noted [92,97]. However, determining the long-term consequences of this phenomenon requires further research.

### 4.2. DXA Matter

BMD performed with a DXA tool are presented as g/cm^3^ areal density. Results depend on changeable factors such as the size of particular parts of examined bone and the positioning of scanned parts according to parallel X-rays. A simple interpretation of expressed BMD values could bring erroneous conclusions. Studies reveal that results could be incomparable considering different sized bones in which the distribution of mineral components varies. Therefore, we propose a new approach, presenting bone density as BMAD g/cm^3^ (Bone mineral apparent density) and IBS, g^2^/cm^4^ (Index of Bone Strength) [98]. Depending on the rotation of bone structures, BMD could change significantly. Bone rebuilds according to forces acting on its cytoskeleton; concave parts (more compressive strengths and overloads) usually have a higher BMD ratio than convex parts. DXA summarizes varieties and averages results [99,100,101]. New models of mineral parts distribution connect density with anthropometric measurements in pediatric patients, such as height, weight, age, sex, race, fat tissue percentage and stage of maturity, to set a better comparative standard, particularly for children with precise anthropometric values. Moreover, some statistical models of femoral bone were created to evaluate the risk of potential fracture [102]. These models are changeable with regards to size and BMD. CT scans and BMD results were fused to compare fractured bones with a control group without fracture. The presented models have more powerful predictive aspects than the typical interpretation of BMD results [103]. A visualization of particular parts of the femoral bone, such as the intertrochanteric region and the femoral neck in cross-sected areas (CSA), reveals trabecular and cortical bone interplay, setting the endurance of the bone. A wider CSA of trabecular bone connects with a relatively higher risk of fracture in the area under investigation. Additionally, the length of the femur and axis profile correlates with fracture appearance. The incidence is higher in patients with a larger angle between neck and shaft [104]. This investigation has proven that the proposed model captures changes in BMD more sensitively than DXA, and is accordingly able to predict future fractures. An elegant comparison performed by Leslie et al. concluded that the proximal part of femoral bone BMD measurements are superior than lumbar spine BMD evaluation for the prediction of global fracture. In an investigated group of 16,505 women, the femoral bone was the best part of the skeleton to calculate appropriate fracture risk [105]. Lobres-Linares et al. propose a modelling tool to simulate the cortical bone microstructure. The presented method aids the creation of bio-mimetic bony materials with adequate strength and endurance, based on BMD distribution in healthy bone tissue [106]. Moreover, Kytýř et al. tested mechanically artificial materials and structures mimicking bone tissue printed using 3D technology as potential agents used in the repairing of trabecular bone injuries and diseases [107]. All of the methods presented above attempt to improve the evaluation of the bio-distribution of mineral components in bone tissue. Most of them mark the insufficiency of the DXA method in interpreting the results properly. We encourage a search for other revolutionary methods [108,109,110,111].

## 5. Conclusions

This study reveals the bio-distribution of hydroxyapatite and another mineral molecules in the femoral neck, systematizing the knowledge of density in this area. The femoral neck, as a region susceptible to trauma, should be given insightful consideration. The average results presented with the DXA method are insufficient in some cases. We believe that the development of the presented method will improve the interpretation of DXA results in patients with density and bio-distribution disorders. Hydroxyapatite studies bring some new possibilities in bone disorders therapy. To draw more specific conclusions on the therapy applied to individual patients, we need to determine the correct orientation of the bone from the resulting density and document the trends in the density distribution change.

## Figures and Tables

**Table 1:** Statistics of the local extremes of brightness curves.

	Curve (Patient)	Maximum 1	Minimum 1	Maximum 2	Relative Decrease
Group 1	A	18.53	10.84	12.20	11%
	B	18.07	6.83	9.15	25%
	D	16.14	11.19	12.78	12%
	F	19.47	11.76	12.94	9%
	Group 1 average	18.05	10.16	11.77	14.5%
	Group 1 standard deviation	1.401	2.251	1.771	
Group 2	C	16.00	9.58	9.87	3%
	E	15.42	8.72	9.11	4%
	G	14.85	4.55	4.55	0%
	H	18.71	11.54	11.72	2%
	Group 2 average	16.25	8.60	8.81	2.2%
	Group 2 standard deviation	1.709	2.942	3.045	

## Data Availability

Data sharing is not applicable to this article.

## References

[1] Consensus Development Conference (1993). Diagnosis, prophylaxis, and treatment of osteoporosis. Am. J. Med..

[2] Cameron J.R., Sorenson G. (1963). Measurements of bone mineral in vivo: An improved method. Science.

[3] Roux C., Briot K. (2017). Current role for bone absorptiometry. Jt. Bone Spine.

[4] National Osteoporosis Foundation (2010). Clinician’s Guide to Prevention and Treatment of Osteoporosis.

[5] Watts N.B., Bilezikian J.P., Camacho P.M., Greenspan S.L., Harris S.T., Hodgson S.F., Kleerekoper M., Luckey M.M., McClung M.R., Pollack R.P. (2010). American Association of Clinical Endocrinologists medical guidelines for clinical practice for the diagnosis and treatment of postmenopausal osteoporosis. Endocr. Prac..

[6] Bonnick S.L. (2004). Bone Densitometry in Clnical Practice.

[7] Khan A.A., Colquhoun A., Hanley D.A., Jankowski L.G., Josse R.G., Kendler D.L., Lentle B., Leslie W.D., Lewiecki E.M., O’Neill E. (2007). Standards and guidelines for technologists performing central dual-energy X-ray absorptiometry. J. Clin. Densitom..

[8] Leslie W.D. (2008). Factors affecting short-term bone density precision assessment and the effect on patient monitoring. J. Bone Miner. Res..

[9] Leslie W.D., Moayyeri A., Sadatsafavi M., Wang L. (2007). A new approach for quantifying change and test precision in bone densitometry. J. Clin. Densitom..

[10] Baniak N., Grzybowski S., Olszynski W.P. (2014). Dual-energy X-ray absorptiometry scan autoanalysis vs manual analysis. J. Clin. Densitom..

[11] Rosenthall L. (2004). Range of change of measured BMD in the femoral neck and total hip with rotation in women. J. Bone Miner. Metab..

[12] Olszta M.J., Cheng X., Jee S.S., Kumar R., Kim Y.-Y., Kaufman M.J., Douglas E.P., Gower L.B. (2007). Bone structure and formation: A new perspective. Mat. Sci. Eng. R.

[13] Cengiz B., Gokce Y., Yildiz N., Aktas Z., Calimli A. (2008). Synthesis and characterization of hydroxyapatite nanoparticles. Colloids Surf. A Physicochem. Eng. Asp..

[14] Bassi M.A., Lopez M.A., Confalone L., Carinci F. (2015). Hydraulic Sinus Lift Technique in Future Site Development: Clinical and Histomorphometric Analysis of Human Biopsies. Implant. Dent..

[15] Ma P.X. (2004). Scaffolds for tissue fabrication. Mater. Today.

[16] Mann S. (1988). Molecular recognition in biomineralization. Nature.

[17] Dorozhkin S.V. (2015). Calcium orthophosphate bioceramics. Ceram. Int..

[18] Rabiei M., Palevicius A., Monshi A., Nasiri S., Vilkauskas A., Janusas G. (2020). Comparing Methods for Calculating Nano Crystal Size of Natural Hydroxyapatite Using X-Ray Diffraction. Nanomaterials.

[19] Dorozhkin S.V. (2016). Multiphasic calcium orthophosphate (CaPO_4_) bioceramics and their biomedical applications. Ceram. Int..

[20] Turon P., Del Valle L., Aleman C., Puiggali J. (2017). Biodegradable and Biocompatible Systems Based on Hydroxyapatite Nanoparticles. Appl. Sci..

[21] Noor Z. (2013). Nanohydroxyapatite application to osteoporosis management. J. Osteoporos..

[22] Chen F., Wang Z., Lin C. (2002). Preparation and characterization of nano-sized hydroxyapatite particles and hydroxyapatite/chitosan nano-composite for use in biomedical materials. Mater. Lett..

[23] Koutsopoulos S. (2002). Synthesis and characterization of hydroxyapatite crystals: A review study on the analytical methods. J. Biomed. Res..

[24] Zhou H., Lee J. (2011). Nanoscale hydroxyapatite particles for bone tissue engineering. Acta Biomater..

[25] Wang F., Li M., Lu Y., Qi Y., Liu Y. (2006). Synthesis and microstructure of hydroxyapatite nanofibers synthesized at 37 °C. Mater. Chem. Phys..

[26] Sun F., Zhou H., Lee J. (2011). Various preparation methods of highly porous hydroxyapatite/polymer nanoscale biocomposites for bone regeneration. Acta Biomater..

[27] Wahl D.A., Czernuszka J.T. (2006). Collagen-hydroxyapatite composites for hard tissue repair. Eur. Cell Mater..

[28] Junqueira L.C., José C., Foltin J., Lebowitz H., Boyle P.J. (2003). Basic Histology, Text & Atlas.

[29] Parhi P., Ramanan A., Ray A.R. (2004). A convenient route for the synthesis of hydroxyapatite through a novel microwave-mediated metathesis reaction. Mater. Lett..

[30] Marchi J., Dantas A.C.S., Greil P. (2007). Influence of Mg-substitution on the physicochemical properties of calcium-phosphate powders. Mater. Res. Bull..

[31] Olaechea A., Mendoza-Azpur G., O’valle F., Padial-Molina M., Martin-Morales N., Galindo-Moreno P. (2019). Biphasic hydroxyapatite and ß-tricalcium phosphate biomaterial behavior in a case series of maxillary sinus augmentation in humans. Clin Oral Impl. Res..

[32] De Godoy R.F., Hutchens S., Campion C., Blunn G. (2015). Silicate-substituted calcium phosphate with enhanced strut porosity stimulates osteogenic differentiation of human mesenchymal stem cells. J. Mater. Sci. Mater. Med..

[33] Kojima S., Nakamura H., Lee S., Nagata F., Kato K. (2019). Hydroxyapatite Formation on Self-Assembling Peptides with Differing Secondary Structures and Their Selective Adsorption for Proteins. Int. J. Mol. Sci..

[34] Groppo M.F., Caria P.H., Freire A.R., Figueroba S.R., Ribeiro-Neto W.A., Bretas R.E.S., Prado F.B., Haiter-Neto F., Aguiar F.H.B., Rossi A.C. (2017). The effect of a hydroxyapatite impregnated PCL membrane in rat subcritical calvarial bone defects. Arch Oral Biol..

[35] Sadeghi M., Bakhshandeh B., Dehghan M.M., Mehrnia M.R., Khojasteh A. (2016). Functional synergy of anti-mir221 and nanohydroxyapatite scaffold in bone tissue engineering of rat skull. J. Mater Sci. Mater Med..

[36] Luo Z.B., Zhang Q.-B., Zhang Z.-Q., Chen D., Yan W.-X., Li K.-F., Chen Y. (2013). Performance of coralline hydroxyapatite in sinus floor augmentation: A retrospective study. Clin. Oral Investig..

[37] Dent C.E., Davies I.J.T. (1980). Calcium metabolism in bone disease: Effects of treatment with microcrystalline calcium hydroxyapatite compound and dihydrotachysterol. J. R. Soc. Med..

[38] Chu M., Liu G. (2005). Preparation and characterization of hydroxyapatite/liposome core-shell nanocomposites. Nanotechnology.

[39] Grenho L., Salgado C.L., Fernandes M.H.F., Monteiro F.J., Ferraz M.P. (2015). Antibacterial activity and biocompatibility of three-dimensional nanostructured porous granules of hydroxyapatite and zinc oxide nanoparticles—An in vitro and in vivo study. Nanotechnology.

[40] Salehi M., Naseri-Nosar M., Ebrahimibarough S., Nourani M., Vaez A., Farzamfar S., Ai J. (2018). Regeneration of sciatic nerve crush injury by a hydroxyapatite nanoparticle-containing collagen type I hydrogel. J. Physiol. Sci..

[41] Chaves M.D., Nunes L.S.D.S., de Oliveira R.V., Holgado L.A., Filho H.N., Matsumoto M.A., Ribeiro D. (2012). Bovine hydroxyapatite (Bio-Oss) induces osteocalcin, RANK-L and osteoprotegerin expression in sinus lift of rabbits. J. Craniomaxillofac. Surg..

[42] Qiu H., Yang J., Kodali P., Koh J., Ameer G.A. (2006). A citric acid-based hydroxyapatite composite for orthopedic implants. Biomaterials.

[43] Jafari S. (2015). Application of hydroxyapatite nanoparticle in the drug delivery systems. J. Mol. Pharm. Org. Process Res..

[44] De Jong W.H., Borm P.J. (2008). Drug delivery and nanoparticles: Applications and hazards. Int. J. Nanomed..

[45] Peng H., Wang J., Lv S., Wen J., Chen J.F. (2015). Synthesis and characterization of hydroxyapatite nanoparticles prepared by a high-gravity precipitation method. Ceram. Int..

[46] Song J.M., Shin S.H., Kim Y.D., Lee J.Y., Baek Y.J., Yoon S.Y., Kim H.S. (2014). Comparative study of chitosan/fibroin–hydroxyapatite and collagen membranes for guided bone regeneration in rat calvarial defects: Micro-computed tomography analysis. Int. J. Oral. Sci..

[47] da Silva Brum I., de Carvalho J.J., da Silva Pires J.L., de Carvalho M.A.A., Dos Santos L.B.F., Elias C.N. (2019). Nanosized hydroxyapatite and β-tricalcium phosphate composite: Physico-chemical, cytotoxicity, morphological properties and in vivo trial. Sci. Rep..

[48] Faeda R.S., Tavares H.S., Sartori R., Guastaldi A.C., Marcantonio E. (2009). Biological performance of chemical hydroxyapatite coating associated with implant surface modification by laser beam: Biomechanical study in rabbit tibias. J. Oral Maxillofac. Surg..

[49] Govindaraj D., Rajan M., Munusamy M.A., Alarfaj A.A., Sadasivuni K.K., Kumar S.S. (2017). The synthesis, characterization and in vivo study of mineral substituted hydroxyapatite for prospective bone tissue rejuvenation applications. Nanomedicine.

[50] Schroeder A., Heller D.A., Winslow M.M., Dahlman J.E., Pratt G.W., Langer R., Jacks T., Anderson D.G. (2012). Treating metastic cancer with nanotechnology. Nat. Rev. Cancer.

[51] Rossi A.L., Longuinho M., Tanaka M.N., Farina M., Borojevic R., Rossi A.M. (2018). Intracellular pathway and subsequent transformation of hydroxyapatite nanoparticles in the SAOS-2 osteoblast cell line. J. Biomed. Mater. Res. A.

[52] Qayoom I., Teotia A.K., Kumar A. (2019). Nanohydroxyapatite Based Ceramic Carrier Promotes Bone Formation in a Femoral Neck Canal Defect in Osteoporotic Rats. Biomacromolecules.

[53] Raina D.B., Isaksson H., Hettwer W., Kumar A., Lidgren L., Tägil M. (2016). A Biphasic Calcium Sulphate/Hydroxyapatite Carrier Containing Bone Morphogenic Protein-2 and Zoledronic Acid Generates Bone. Sci. Rep..

[54] Gao M., Gao W., Papadimitriou J.M., Zhang C., Gao J., Zheng M. (2018). Exosome the Enigmatic Regulators of Bone Homeostasis. Bone Res..

[55] Teotia A.K., Raina D.B., Singh C., Sinha N., Isaksson H., Tägil M., Lidgren L., Kumar A. (2017). Nano-Hydroxyapatite Bone Substitute Functionalized with Bone Active Molecules for Enhanced Cranial Bone Regeneration. ACS Appl. Mater. Interfaces.

[56] Sahana H., Khajuria D.K., Razdan R., Mahapatra D.R., Bhat M.R., Suresh S., Rao R.R., Mariappan L. (2013). Improvement in Bone Properties by Using Risedronate Adsorbed Hydroxyapatite Novel Nanoparticle Based Formulation in a Rat Model of Osteoporosis. J. Biomed. Nanotechnol..

[57] Rabiei M., Palevicius A., Ebrahimi-Kahrizsangi R., Nasiri S., Vilkauskas A., Janusas G. (2021). New Approach for Preparing In Vitro Bioactive Scaffold Consisted of Ag-Doped Hydroxyapatite + Polyvinyltrimethoxysilane. Polymers.

[58] Zhang X., Zhu L., Lv H., Cao Y., Liu Y., Xu Y., Ye W., Wang J. (2012). Repair of rabbit femoral condyle bone defects with injectable nanohydroxyapatite/chitosan composites. J. Mater. Sci..

[59] Noor Z., Sumitro S.B., Hidayat M., Rahim A.H., Sabarudin A., Umemura T. (2012). Atomic mineral characteristics of Indonesian osteoporosis by high-resolution inductively coupled plasma mass spectrometry. Sci. World J..

[60] Yang F., Dong W.-J., He F.-M., Wang X.-X., Zhao S.-F., Yang G.-L. (2012). Osteoblast response to porous titanium surfaces coated with zinc-substituted hydroxyapatite. Oral Surg. Oral Med. Oral Pathol. Oral Radiol. Endodontology.

[61] Chang M.C. (2008). Fluoride incorporation in hydroxyapatite/gelatin nanocomposite. J. Mater. Sci..

[62] Li D.-H., Lin J., Lin D.-Y., Wang X.-X. (2011). Synthesized silicon-substituted hydroxyapatite coating on titanium substrate by electrochemical deposition. J. Mater. Sci..

[63] Xie M., Olderøy M.Ø., Andreassen J.-P., Selbach S.M., Strand B.L., Sikorski P. (2010). Alginate-controlled formation of nanoscale calcium carbonate and hydroxyapatite mineral phase within hydrogel networks. Acta Biomater..

[64] Fabbri P., Bondioli F., Messori M., Bartoli C., Dinucci D., Chiellini F. (2010). Porous scaffolds of polycaprolactone reinforced with in situ generated hydroxyapatite for bone tissue engineering. J. Mater. Sci..

[65] Ni G.-X., Huang G., Lu W.W., Pan H.-B. (2012). The effect of strontium incorporation into hydroxyapatite on their physical and biological propertoes. J. Biomed. Mater. Res. B.

[66] Wang J., Nonami T., Yubata K. (2008). Syntheses, structures and photophysical properties of iron containing hydroxyapatite prepared by a modified pseudo-body solution. J. Mater. Sci..

[67] Sözen T., Özışık L., Basaran N.C. (2017). An Overview and Management of Osteoporosis. Eur. J. Rheumatol..

[68] Duncan E.L., Brown M.A. (2008). Genetic studies in osteoporosis—The end of the beginning. Arthritis Res. Ter..

[69] Song Y., Liebschner M.A.K., Gunaratne G.H. (2004). A study of age-related architectural changes that are most damaging to bones. Biophys. J..

[70] Walters S., Khan T., Ong T., Sahota O. (2017). Fracture liaison services: Improving outcomes for patients with osteoporosis. Clin. Interv. Aging.

[71] Brandao C.M.R., Lima M.G., da Silva A.L., Silva G.D., Guerra A.A.G., De Assis Acurcio F. (2008). Treatment of postmenopausal osteoporosis in women: A systematic review. Cad. De Saude Publica.

[72] Huang Q.-Y., Kung A.W.C. (2006). Genetics of osteoporosis. Mol. Genet. Metab..

[73] Office of the Surgeon General (US) (2004). Bone Health and Osteoporosis.

[74] Qaseem A., Forciea M.A., McLean R.M., Denberg T.D. (2017). Treatment of Low Bone Density or Osteoporosis to Prevent Fractures in Men and Women: A Clinical Practice Guideline Update from the American College of Physicians. Ann. Intern. Med..

[75] Noor Z. (2011). Substitution and Incorporation of Atomic Minerals, Hydroxyapatite Crystale and Microstructure of Osteoporosis Bone. Ph.D. Thesis.

[76] Castelo-Branco C., Ciria-Recasens M., Cancelo-Hidalgo M.J., Palacios S., Haya-Palazuelos J., Carbonell-Abelló J., Blanch-Rubió J., Martínez-Zapata M.J., Manasanch J., Pérez-Edo L. (2009). Efficacy of ossein-hydroxyapatite complex compared with calcium carbonate to prevent bone loss: A meta-analysis. Menopause.

[77] Castelo-Branco C., Pons F., Vicente J.J., Sanjuan A., Vanrell J.A. (1999). Preventing postmenopausal bone loss with osseinhydroxyapatite compounds: Results of a two-year, prospective trial. J. Reprod. Med. Obstet. Gynecol..

[78] Ciria-Recasens M., Blanch-Rubio J., Coll-Batet M., del Pilar Lisbona-Pérez M., Díez-Perez A., Carbonell-Abelló J., Manasanch J., Pérez-Edo L. (2011). Comparison of the effects of ossein-hydroxyapatite complex and calcium carbonate on bone metabolism in women with senile osteoporosis: A randomized, open-label, parallel-group, controlled, prospective study. Clin. Drug Investig..

[79] Guha A.K., Singh S., Kumaresan R., Nayar S., Sinha A. (2009). Mesenchymal cell response to nanosized biphasic calcium phosphate composites. Colloids Surf. B Biointerfaces.

[80] Han Y., Li S., Cao X., Yuan L., Wang Y., Yin Y., Qiu T., Dai H., Wang X. (2014). Different inhibitory effect and mechanism of hydroxyapatite nanoparticles on normal cells and cancer cells in vitro and in vivo. Sci. Rep..

[81] Russmueller G., Winkler L., Lieber R., Seemann R., Pirklbauer K., Perisanidis C., Kapeller B., Spassova E., Halwax E., Macfelda K. (2017). In vitro effects of particulate bone substitute materials on the resorption activity of human osteoclasts. Eur. Cell Mater..

[82] Yin M., Yin Y., Han Y., Dai H., Li S. (2014). Effects of uptake of hydroxyapatite nanoparticles into hepatoma cells on cell adhesion and proliferation. J. Nanomater..

[83] Liao C., Zhou J. (2014). Replica exchange molecular dynamics simulation of basic fibroblast growth factor adsorption on hydroxyapatite. J. Phys. Chem. B.

[84] Lee J., Yun H. (2014). Hydroxyapatite-containing gelatin/chitosan microspheres for controlled release of lysozyme and enhanced cytocompatibility. J. Mater. Chem. B.

[85] Nathanael A.J., Yuvakkumar R., Hong S.I., Oh T.H. (2014). Novel zirconium nitride and hydroxyapatite nanocomposite coating: Detailed analysis and functional properties. ACS Appl. Mater. Interfaces.

[86] McAllister B.S., Haghighat K. (2007). Bone augmentation techniques. J. Periodontol..

[87] Johari B., Ahmadzadehzarajabad M., Azami M., Kazemi M., Soleimani M., Kargozar S., Hajighasemlou S., Farajollahi M.M., Samadikuchaksaraei A. (2016). Repair of rat critical size calvarial defect using osteoblast-like and umbilical vein endothelial cells seeded in gelatin/hydroxyapatite scaffolds. J. Biomed. Mater. Res. A.

[88] Ferraz M.P., Monteiro F.J., Serro A.P., Saramago B., Gibson I.R., Santos J.D. (2001). Effect of chemical composition on hydrophobicity and zeta potential of plasma sprayed HA/CaO-P_2_O_5_ glass coatings. Biomaterials.

[89] Tautzenberger A., Lorenz S., Kreja L., Zeller A., Musyanovych A., Schrezenmeier H., Katharina L., Volker M., Anita I. (2010). Effect of functionalized fluorescence-labelled nanoparticles on mesenchymal stem cell differentiation. Biomaterials.

[90] Tautzenberger A., Kreja L., Zeller A., Lorenz S., Schrezenmeier H., Mailänder V., Katharina L., Anita I. (2011). Direct and indirect effects of functionalized fluorescence-labelled nanoparticles on human osteoclast formation and activity. Biomaterials.

[91] Maia A.L., Cavalcante C.H., Souza M.G., Ferreira Cde A., Rubello D., Chondrogiannis S., Cardoso V.N., Ramaldes G.A., Barros A.L., Soares D.C. (2016). Hydroxyapatite nanoparticles: Preparation, characterization, and evaluation of their potential use in bone targeting: An animal study. Nucl. Med. Commun..

[92] Lopes S.C.A., Novais M.V.M., Teixeira C.S., Honorato-Sampaio K., Pereira M.T.P., Ferreira L.A.M., Braga F.C., Oliveira M.C. (2013). Preparation, physicochemical characterization, and cell viability evaluation of long-circulating and pH-sensitive liposomes containing ursolic acid. Biomed. Res. Int..

[93] Uskokovic V., Odsinada R., Djordjevic S., Habelitz S. (2011). Dynamic light scattering and zeta potential of colloidal mixtures of amelogenin and hydroxyapatite in calcium and phosphate rich ionic milieus. Arch. Oral. Biol..

[94] Solberg B.D., Gutow A.P., Baumgaertner M.R. (1999). Efficacy of gentamycinimpregnated resorbable hydroxyapatite cement in treating osteomyelitis in a rat model. J. Orthop. Trauma.

[95] Korkusuz F., Uchida A., Shinto Y., Araki N., Inoue K., Ono K. (1993). Experimental implant-related osteomyelitis treated by antiotic-calcium hydroxyapatite ceramic composites. J. Bone Joint Surg. Br..

[96] Carcia C.R., Scibek J.S. (2013). Causation and management of calcific tendonitis and periarthritis. Curr. Opin. Rheumatol..

[97] De Barros A.L.B., Chacko A., Mikitsh J.L., Al-Zaki A., Saboury B., Tsourkas A., Alavi A. (2013). Assessment of global cardiac uptake of radiolabeled iron oxide nanoparticles in apolipoprotein-E-deficient mice. Mol. Imag. Biol..

[98] Carter D.R., Bouxsein M.L., Marcus R. (1992). New approaches for interpreting projected bone densitometry data. J. Bone Miner. Res..

[99] Routh R.H., Rumancik S., Pathak R.D., Burshell A.L., Nauman E.A. (2005). The relationship between bone mineral density and biomechanics in patients with osteoporosis and scoliosis. Osteoporos. Int..

[100] Rumancik S., Routh R.H., Pathak R.D., Burshell A.L., Nauman E.A. (2005). Assessment of bone quantity and distribution in adult lumbar scoliosis: New dual-energy x-ray absorptiometry methodology and analysis. Spine.

[101] Cheng J.C., Sher H.L., Guo X., Hung V.W., Cheung A.Y. (2001). The effect of vertebral rotation of the lumbar spine on dual energy X-ray absorptiometry measurements: Observational study. Hong Kong Med. J..

[102] Short D.F., Zemel B.S., Gilsanz V., Kalkwarf H.J., Lappe J.M., Mahboubi S., Oberfield S.E., Shepherd J.A., Winer K.K., Hangartner T.N. (2011). Fitting of bone mineral density with consideration of anthropometric parameters. Osteoporos. Int..

[103] Whitmarsh T., Fritscher K.D., Humbert L., Barquero L.M.D.R., Roth T., Kammerlander C., Blauth M., Schubert R., Frangi A.F., Fichtinger G., Martel A., Peters T. (2011). A Statistical Model of Shape and Bone Mineral Density Distribution of the Proximal Femur for Fracture Risk Assessment. Medical Image Computing and Computer-Assisted Intervention—MICCAI 2011.

[104] Ito M., Wakao N., Hida T., Matsui Y., Abe Y., Aoyagi K., Uetani M., Harada A. (2010). Analysis of hip geometry by clinical CT for the assessment of hip fracture risk in elderly Japanese women. Bone.

[105] Leslie W.D., Lix L.M., Tsang J.F., Caetano P.A. (2007). Manitoba Bone Density Program. Single-Site vs Multisite Bone Density Measurement for Fracture Prediction. Arch. Intern. Med..

[106] Robles-Linares J.A., Ramírez-Cedillo E., Siller H.R., Rodríguez C.A., Martínez-López J.I. (2019). Parametric Modeling of Biomimetic Cortical Bone Microstructure for Additive Manufacturing. Materials.

[107] Kytýř D., Zlámal P., Koudelka P., Fíla T., Krčmářová N., Kumpová I., Vavřík D., Gantar A., Novakcd S. (2017). Deformation analysis of gellan-gum based bone scaffold using on-the-fly tomography. Mater. Des..

[108] Pathi S.P., Lin D.D.W., Dorvee J.R., Estroff L.A., Fischbach-Teschl C. (2011). Hydroxyapatite nanoparticle-containing scaffolds for the study of breast cancer bone metastasis. Biomaterials.

[109] Gomi K., Lowenberg B., Shapiro G., Davies J.E. (1993). Resorption of sintered synthetic hydroxyapatite by osteoclasts in vitro. Biomaterials.

[110] Redey S.A., Razzouk S., Rey C., Bernache-Assollant D., Leroy G., Nardin M., Cournot G. (1999). Osteoclast adhesion and activity on synthetic hydroxyapatite, carbonated hydroxyapatite, and natural calcium carbonate: Relationship to surface energies. J. Biomed. Mater..

[111] Raina D.B., Larsson D., Mrkonjic F., Isaksson H., Kumar A., Lidgren L., Tagil M. (2018). Gelatin- Hydroxyapatite- Calcium Sulphate Based Biomaterial for Long Term Sustained Delivery of Bone Morphogenic Protein-2 and Zoledronic Acid for Increased Bone Formation: In-Vitro and in-Vivo Carrier Properties. J. Control. Release.

